# Epigenetics of Indolent Lymphoma and How It Drives Novel Therapeutic Approaches—Focus on EZH2-Targeted Drugs

**DOI:** 10.1007/s11912-021-01076-0

**Published:** 2021-05-03

**Authors:** Jemma Longley, Peter W. M. Johnson

**Affiliations:** University of Southampton, Southampton, UK

**Keywords:** Follicular lymphoma, Epigenetics, EZH2 inhibitors, Tazematostat

## Abstract

**Purpose of Review:**

Epigenetic modifier gene mutations are common in patients with follicular lymphoma. Here we review the pathogenesis of these mutations and how they are targeted by epigenetic drugs including EZH2 inhibitors in both mutated and wild-type disease.

**Recent Findings:**

The use of EZH2 inhibitor tazematostat in early phase clinical trials has proved encouraging in the treatment of follicular lymphoma harbouring an EZH2 mutation; however, responses are also seen in patients with wild-type disease which is partially explained by the off target effects of EZH2 inhibition on immune cells within the tumour microenvironment.

**Summary:**

Further studies incorporating prospective molecular profiling are needed to allow stratification of patients at both diagnosis and relapse to further our understanding of how epigenetic modifier mutations evolve over time. The use of tazematostat in combination or upfront in patients with an EZH2 mutation remains unanswered; however, given durable responses, ease of oral administration, and tolerability, it is certainly an attractive option.

## Introduction

Indolent lymphomas mostly consist of low-grade lymphomas of B cell origin, classified according to their pathological and clinical features. Follicular lymphoma (FL) is the most common type of indolent B cell non-Hodgkin lymphoma (NHL) derived from germinal centre (GC) B cells. Its clinical course is complex and characterised by multiple relapses which can eventually lead to treatment refractory disease or transformation into a more aggressive form, most commonly diffuse large B cell lymphoma (DLBCL) in 30% of cases. In both refractory and transformed disease, patient survival outcomes are poor and treatment options limited outside the clinical trial setting. There is an urgent need within this patient population to develop new approaches based on our increased understanding of the mutational landscape of FL derived from next-generation sequencing. Epigenetic modifier gene mutations are highly prevalent in FL, hijacking large transcriptional programmes within the normal B cell to maintain tumour growth and survival [1]. These include loss of function mutations in CREB-binding protein (CREBBP) and E1A-binding protein p300 (EP300) that encode for histone acetylation enzymes and gain of function mutations in enhancer of zeste homolog 2 (EZH2) which encodes for a methylation enzyme resulting in modification of chromatin state and a subsequent change in transcription pattern [2]. During the last 10 years, drugs targeting these epigenetic regulators have been trialled with varied success; however, the use of EZH2 inhibitors especially in those patients with an activating mutation has proved encouraging in early phase clinical trials [3•]. This article will detail the epigenetic landscape of FL and how this is exploited by new targeted drugs with a focus on EZH2 inhibitors both as a single agent and in combination with other immunochemotherapies.

## Mutational Landscape of Follicular Lymphoma

Epigenetics is the mechanism by which changes in gene expression occur without alterations in a DNA sequence. This can be achieved by histone remodelling, DNA methylation, and post transcriptional repression by non-coding RNAs. The alteration of chromatin state to allow access for RNA transcriptional machinery is mediated by histone acetylation and methylation. Histone methylation is regulated by histone methyl transferases (HMTs) and histone demethylases (HDMTs) which can either silence or activate gene transcription depending on the number and site of methyl groups added to a histone protein. Histone acetylation is controlled by histone acetyltransferases (HATs) and histone deacetylases (HDACs) by the addition or removal of an acetyl group from a lysine amino acid at the N-terminus of a histone protein, respectively. Histone acetylation results in reduced affinity for DNA binding promoting gene transcription by the dissociation and unpacking of DNA from its histone proteins [4].

Normal B cells undergo somatic hypermutation of immunoglobulin heavy and light variable chain regions within the lymph node germinal centre (GC) that leads to increased antigen affinity. This complex process of B cell maturation is controlled by transcription factors capable of achieving rapid epigenetic reprogramming in response to signals from the GC microenvironment via histone modifying enzymes that alter chromatin structure [5]. Within the GC, normal B cells bind antigen presented by follicular dendritic cells (FDCs), and those with highest affinity form immune complexes with follicular T-helper cells (TFHs) via MHC class II stimulating B cell terminal differentiation [6]. Genetic mutations encoding for transcription factors, histone, and chromatin-modifying enzymes in this context have been detected in patients with FL, altering the epigenetic landscape and modifying immune cell interactions within the microenvironment to promote tumour growth and survival [7••]. Targeting these epigenetic modifier mutations may establish the normal B cell transcriptional programme by restoring acetylation and methylation levels and subsequent chromatin architecture. Table 1 shows the most common epigenetic mutations in FL and their frequency.

**Table 1:** Epigenetic modifier gene mutations in FL

Gene name	Biological function	Mutation	Mutational frequency
KMT2D	H3K4 methyltransferase	Loss of function	72%
CREBBP	Lysine acetyltransferase	Loss of function	65%
EZH2	H3K27 methyltransferase	Gain of function	25%
EP300	Lysine acetyltransferase	Loss of function	15%
HIST1H1B-E	Histone linker	Unknown	14%
KMT2C	H3K4 methyltransferase	Loss of function	13%
ARID1A	SWI/SNF component	Unknown	11%
SMARCA4	SWI/SNF component	Loss of function	1%

*H3* histone 3, *K27 K18 K4* number sequence of amino acid lysine residue, *KMT2D* lysine (K)-specific methyltransferase 2D, *CREBBP* CREB-binding protein, *EZH2* enhancer of zeste homolog 2, *EP300* E1A-binding protein p300, *HIST1H1B* histone 1 family genes B-E, *KMT2C* lysine (K)-specific methyltransferase 2C, *ARDIA1A* AT-rich interactive domain 1A, *SMARCA4* SWI/SNF-related, matrix-associated, actin-dependent regulator of chromatin, subfamily A, member 4, *SWI/SNF* switch/sucrose non-fermentable complex

Linker histone H1 proteins facilitate large-scale condensation of chromatin making it inaccessible for transcriptional machinery and by the recruitment of transcriptional repressors. Mutations primarily in alleles H1C and H1E are detected in both NHL (30–40%) and Hodgkin lymphoma (50%), driving a change genomic architecture and relaxation of chromatin state. This in turn leads to the expression of previously silenced stem cell like genes involved in early development. This was demonstrated in a BCL2 lymphoma murine model in mice with H1C and H1E deficiency [8]. These mice had reduced survival and increased infiltration of the liver and lungs with malignant B cells compared with controls. RNA analysis of these B cell lymphomas showed the enrichment for mesenchymal-transition and stem cell genes in both heterozygous and homozygous mice, resulting in B cell lymphomas with enhanced self-renewal properties leading to more aggressive disease.

**Fig. 1:**
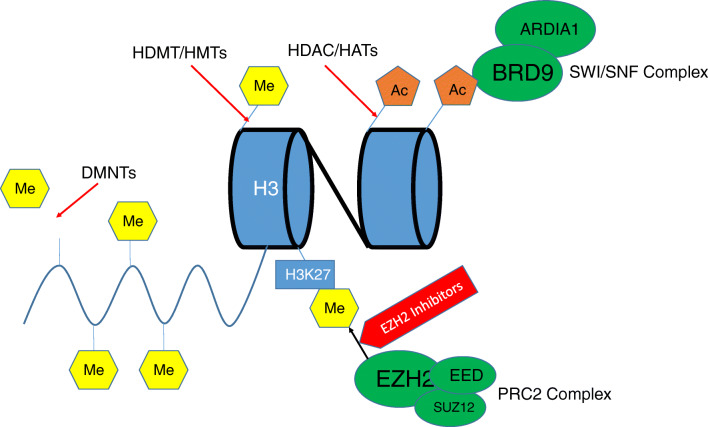
The elements of epigenetic regulation indicating the control of histone methylation and acetylation

EZH2 catalyses the trimethylation of histone H3 at lysine position 27 (H3K27me3) as part of the polycomb repressive complex (PRC2), epigenetically maintaining transcriptional repression of target genes that negatively regulate the cell cycle and terminal differentiation [9]. As cells differentiate, EZH2 activity is reduced by activation of the switch/sucrose non-fermentable (SNF/SWI) multi-chromatin remodelling complex which acts in opposition to PCR2 activity, promoting terminal differentiation (Fig. 1) [7••]. Gain of function EZH2 mutations are present in up to 25% of patients with FL resulting in higher levels of H3K27me3 activity and likely represent an early clonal event in the development of the disease [10]. This was investigated by Bodor et al. by analysing EZH2 in serial FL biopsies. Mutations were clustered to 3 codons, Y646 (most common), A682, and A692, which were mainly clonal in nature and stable throughout disease progression [10]. This may reflect such an early transformation event that EZH2 is no longer required for tumour cell growth beyond the in situ stage and therefore redundant in terms cell oncological dependence. The inevitable development of sub-clonal populations as a result of selection pressure following different treatments over time limits the conclusions that can be drawn from the interpretation of a single site biopsy. There is a need to further understand each individual’s tumour heterogeneity and how driver mutations within sub-clonal populations evolve over time [11].

EZH2 and H3K27me3 are dysregulated in a number of ways which may account for treatment responses seen in both EZH2 mutated (MT) and wild-type (WT) disease. Loss of function in the tumour suppressor gene SNF5 (also known as SMARCB1, INI1, and BAF47), a subunit of the SNF/SWI complex, results in unopposed EZH2 activity and increased H3K27me3 levels which drive tumour growth in the absence of EZH2 gain of function mutations (Fig. 1) [7••]. In addition, inactivating mutations in the KDM6A gene which codes for the H3K27me3 lysine-specific demethylase 6A results in unopposed methylation and subsequent gene repression [4]. The immunomodulatory effects of EZH2 on cells that make up the tumour microenvironment (TME) are also likely to influence response to treatment in both MT and WT disease. EZH2 activity in tumour cells downregulates MHC class I and class II expression on their cell surface, resulting in immune evasion by a reduction in tumour antigen presentation [10]. The transcriptomic analysis of 347 DLBCL patient samples by Enishi et al. demonstrated that EZH2 mutation enrichment of MHC class I and II negative lymphomas was associated with a reduced T cell infiltrate in subsequent studies in vivo, which was reversed by EZH2 inhibition [12•]. This downregulation of MHC class II also negatively impacts the interaction of B cells with TFH cells in the GC microenvironment leading to reduced antigen presentation and CD40 activation. These EZH2 MT FL cells show an increased association with FDCs and an ability to proliferate independent of TFH signals that were sensitive to FDC blockade in vivo [6]. Therefore reprogramming of the TME with EZH2 inhibitors may restore immunogenicity and prime the immune system prior to treatment with other immunochemotherapies in both MT and WT disease.

T cell maturation and differentiation are inhibited by EZH2 expression, by repressing the transcription of genes involved in the formation of effector and memory functions, thereby maintaining plasticity. In vitro and in vivo studies have demonstrated opposing functions of EZH2 inhibition on T cell development [13]. Reduced CD8 T cell clonal expansion has been demonstrated in EZH2 knockout (KO) mice compared with controls with an impaired ability to secrete proinflammatory cytokines and increased apoptosis following antigen stimulation [14••]. However, further subsequent studies involving melanoma mouse models have not shown any effect of EZH2 inhibition on T cell effector function within the TME [15]. This picture is further complicated by EZH2 function in T regulatory (T reg) cells, an immunosuppressive subset of CD4 T cells which have an important role in the development resistance to immunotherapies. EZH2 stabilises FOX3P expression in T regs which maintains their immunosuppressive function following activation [16]. Therefore EZH2 inhibition can promote an anti-tumour response via increased T cell infiltration and activation, restoration of antigen presentation, and the reversal of T reg immunosuppressive function. The role of EZH2 in myeloid cells within the TME is yet to be fully characterised; however, EZH2 expression may polarise macrophages to a proinflammatory phenotype, and its inhibition reduces their cytotoxic and phagocytic activity. This was demonstrated in an in vivo tumour model where macrophage anti-tumour function was restored by PD1 checkpoint blockade following EZH2 inhibition [17]. This shows the importance of understanding the role of EHZ2 not only in tumour cells but in different immune cells that make up the TME to help determine the best combination and sequencing of therapies for each individual patient’s disease.

CREBBP and EP300 mutations are present in up to 65% of patients with FL and lead to reduced acetyltransferase activity, making DNA less accessible to transcription factors and a reduction in transcriptional elongation normally mediated by the binding of bromodomain containing proteins [7••]. These mutations are also associated with a reduction in MHC class II expression in tumour cells, leading to a reduction in antigen presentation, T cell infiltrate, and effector function [12•]. These epigenetic modifier genes along with KDTM2D are mutated more commonly in FL compared with EZH2; however, their loss of function at the protein level makes them difficult to drug, in contrast to EZH2 inhibition.

## HDAC Inhibitors

The development of first-generation HDAC inhibitors in the treatment relapsed/refractory (r/r) NHL showed only a modest response rate as a single agent in early phase clinical trials; however, the majority of these were in patients with r/r FL where responses were durable despite being slow to respond. Vorinostat is an oral HDAC inhibitor investigated in the phase II trial setting by Ogura et al. in patients with indolent B cell lymphomas including FL, marginal zone lymphomas (MZL), small lymphocytic lymphoma (SLL), and mucosal associated lymphoma tissue type (MALT) [18]. This trial included mutational analysis of CREBBP and EP300 of DNA isolated from archival FFPE material, although it is not clear when tissue was obtained and whether a fresh biopsy was required as part of the screening process. Of the 39 patients enrolled with r/r FL, the overall response rate (ORR) was 49% with a median progression-free survival (PFS) of 20 months [18]. The majority of patients with FL were not high risk according to their FLIPI score (31/39), and all but one patient had a pathological grade of 1–2. This higher ORR when compared to previous studies could be explained by these baseline characteristics in a population of patients that were not heavily pre-treated. Retrospective analysis of CREBBP and EP300 mutations did not correlate with clinical outcome and highlights a need for prospective trials which incorporate molecular analysis of fresh tissue biopsies for stratification of patients prior to commencing therapy [18]. The analysis of serial biopsies is essential to study the evolution of epigenetic changes over time to understand how these are modified in response to different therapies. Despite these limitations, the tolerability of vorinostat was favourable in terms of side effect profile with no treatment-related deaths. The study of mocetinostat in r/r FL showed a more modest benefit, with only 3 out of 26 patients experiencing a complete or partial response of their disease [19]. This drug was less well tolerated, with over 50% of patients experiencing one or more grade 3 toxicities [18]. This may be explained by the HDAC isotype preferentially targeted by different classes of HDAC inhibitors and their differential expression depending on cell type.

## EZH2 Inhibitors

Phase 1 first in human studies in patients with haematological and solid tumours has shown conflicting results in EZH2 WT and MT disease. Tazematostat, an oral EZH2 inhibitor, has progressed to phase II trials following responses observed in 38% of patients with B cell NHL including 5 out of 8 patients with indolent B cell lymphomas (4 FL, 1 MZL) at a maximum tolerated dose (MTD) of 800mg twice daily [3•]. Responses were also seen in patients with solid tumours with loss of function mutations in the SNF5 gene, inactivating SNF/SWI activity resulting in an oncological dependence on EZH2 overexpression [3•]. Skin biopsies were used as a surrogate tissue marker for reduction in trimethylation of H3K27 following tazematostat administration and demonstrated a dose-dependent reduction in H3K27m3 within the stratum spinosum layer of the skin using immunohistochemistry (IHC). The most common treatment-related adverse events were asthenia (33%), nausea (20%), and anorexia (14%) all grade 1–2, but overall tazematostat was well tolerated with a favourable safety profile [3•]. However, a phase 1 study of the intravenous EZH2 inhibitor GSK2816126 showed disappointing results in terms of antitumor activity, with only a partial response lasting 3 cycles in 1 patient with DLBCL, for which EZH2 mutation status was unknown [20]. In terms of indolent lymphomas, 4 patients with FL (3 transformed to DLBCL) and 1 patient with MZL were enrolled, but again mutational status could not be performed due to insufficient material. The pharmacokinetics of GSK2816126 revealed that the MTD of 2400mg did not achieve a sufficient trough concentration for the adjusted IC_50_ in vitro protein inhibition of H3K27 trimethylation with a twice weekly IV dosing schedule, but increasing the number of treatments was considered too burdensome for patients [19]. Two patients with a grade 3–4 rise in the enzyme alanine transferase (ALT) was the dose limiting toxicity (DLT) at 3000mg, precluding further dose escalation that would have been required for clinical efficacy [20]. These off target effects combined with very few patients with indolent lymphomas enrolled may account for the negative results seen in this trial.

Analysis of patients with r/r FL treated with tazematostat in the phase II setting has shown ORR of 74% in patients with EZH2 mutant disease and 34% in patients with EZH2 WT disease [21••]. Archival tissue was analysed for EZH2 mutations in codons identified by Bodor et al. using next-generation sequencing of archival tumour DNA. This cohort of patients had heavily pre-treated disease, with 40% refractory to rituximab therapy. Responses were durable, with the disease often slow to respond, but in 50% of patients, this response was maintained for over 12 months [21••]. These encouraging results led to accelerated approval by the FDA in patients with EZH2 MT r/r FL this year resulting in further ongoing clinical trials to assess the efficacy of tazematostat in combination with other standard therapies. The ease of oral administration for patients especially in those with a performance status (PS) of ≥2 resulting in reduced face to face hospital contact is another factor to consider in light of the recent COVID-19 pandemic.

## Combination Therapies

The combination of vorinostat with three weekly rituximab was studied in a phase II trial setting in patients with relapsed/refractory and newly diagnosed indolent NHL and showed an ORR of 67% [22]. This small study of 30 patients when compared to weekly rituximab alone for 4 weeks in a separate trial was similar in terms of ORR in the r/r setting (41% vorinostat plus rituximab 3 weekly verses 40% rituximab weekly). Therefore despite its favourable toxicity profile in combination, it is difficult to justify its addition in terms of clinical benefit. Dual targeting of HDAC and EZH2 in FL is an obvious avenue to investigate, especially in those patients with concurrent EP100, CREBBP, and EZH2 mutations. Pre-clinical studies have demonstrated increased acetylation and decreased methylation of H3K27 in lymphoma cell lines and prolonged survival in mice treated with this combination compared to single agent therapy, highlighting the need for further investigation in the clinical setting [23].

The combination of tazematostat, lenalidomide, and rituximab is currently being investigated in phase I trials for patients with r/r FL with at least one prior line of therapy including rituximab alone and stratified according to mutational status . This chemotherapy-free approach is an attractive one in patients with FL, given its indolent nature and long disease course. Disappointing results were seen with immune checkpoint blockade combined with tazematostat in patients with majorly refractory DLBCL halting any further investigation, which is unsurprising given the minimal clinical activity of single agent tazematostat in earlier clinical trials and insensitivity to PD1/PDL1 checkpoint blockade within this patient population [24]. Priming of the immune system by EZH2 inhibitors may enhance checkpoint blockade in selected patients with epigenetic mutations in FL, highlighting a need for more careful consideration when combining treatments in patients who ideally need to be stratified prospectively according to mutational status to increase our understanding of who may benefit.

## Conclusions

The downstream effects of epigenetic manipulation on gene expression in both normal and malignant cells are extremely complex. Success in early phase trials of targeted epigenetic therapies such as EZH2 inhibitors in FL can be partially explained by oncogenic dependence on EHZ2 gain of function mutations; however, responses in non-mutated disease are poorly understood. The function of EZH2 in different cell types within the TME has shown that patients may still be able to mount an anti-tumour response due to the effect on antigen presentation and T cell function; however, other cell populations including TAMs are skewed to pro-tumour phenotype as a result of EZH2 inhibition. The evolution of epigenetic changes over time is influenced by the type of upfront treatment regime received and is highlighted by the m7FLIPI score variability in predicting 5-year survival when validated in phase III clinical trials [25]. The prospective molecular profiling with next-generation sequencing techniques at diagnosis and relapse should provide more information on the changing molecular landscape of FL and allow stratification of patients according to the mutational status of their disease. The use of tazematostat in relapsed/refractory FL has shown great promise from results of its phase II trial, with some patients maintaining a durable response of over 2 years [21••]. The question of whether EZH2 inhibition in this group of patients should be added to initial standard immunochemotherapy regimes remains to be investigated; however, given the durable responses seen, it may provide initial long-term disease control without the need for chemotherapy. However, patients with WT disease had only a modest response of 34%, highlighting the need to establish biomarkers which will allow identification of those patients that are most likely to benefit [20]. Trials including combination therapies of EZH2 inhibitors with other immunomodulatory therapies such as lenalidomide plus rituximab are awaited in the r/r FL setting, but checkpoint blockade combination has proved disappointing despite the rationale of priming the TME by reversing immunosuppression with EZH2 inhibitors [24]. Patient selection is key when planning further trials and justification of combining therapies dictated by our understanding of individual tumour biology. It is clear that we are entering an exciting new era of targeted therapies for patients with FL, which will expand as our knowledge of the epigenetic landscape increases. The future identification of biomarkers based on prospective molecular profiling will allow stratification of patients at diagnosis and relapse according to their individual disease.
